# High effectiveness of self-help programs after drug addiction therapy

**DOI:** 10.1186/1471-244X-6-35

**Published:** 2006-08-23

**Authors:** John-Kåre Vederhus, Øistein Kristensen

**Affiliations:** 1 Addiction Unit, Sørlandet Hospital, Kristiansand, Norway

## Abstract

**Background:**

The self-help groups Alcoholics Anonymous (AA) and Narcotics Anonymous (NA) are very well established. AA and NA employ a 12-step program and are found in most large cities around the world. Although many have argued that these organizations are valuable, substantial scepticism remains as to whether they are actually effective. Few treatment facilities give clear recommendations to facilitate participation, and the use of these groups has been disputed. The purpose of this study was to examine whether the use of self-help groups after addiction treatment is associated with higher rates of abstinence.

**Methods:**

One hundred and fourteen patients, 59 with alcohol dependency and 55 with multiple drug dependency, who started in self-help groups after addiction treatment, were examined two years later using a questionnaire. Return rate was 66%. Six (5%) of the patients were dead.

**Results:**

Intention-to-treat-analysis showed that 38% still participated in self-help programs two years after treatment. Among the regular participants, 81% had been abstinent over the previous 6 months, compared with only 26% of the non-participants. Logistic regression analysis showed OR = 12.6, 95% CI (4.1–38.3), p < 0.001, for participation and abstinence.

**Conclusion:**

The study has several methodological problems; in particular, correlation does not necessarily indicate causality. These problems are discussed and we conclude that the probability of a positive effect is sufficient to recommend participation in self-help groups as a supplement to drug addiction treatment.

**Previous publication:**

This article is based on a study originally published in Norwegian:

Kristensen O, Vederhus JK: **Self-help programs in drug addiction therapy. ***Tidsskr Nor Laegeforen *2005, **125**:2798–2801.

## Background

Dependency syndrome due to psychoactive substance use is a complex condition in which the ability to control one's own behaviour in relation to the use of the drug has a central dimension. Self-help groups represent an interesting possibility for maintaining sobriety. Alcoholics Anonymous (AA) is the best known. The movement started in 1935 [1]. Narcotics Anonymous (NA) sprang from the AA movement 20 years later. Narcotics Anonymous (NA) implements almost the same program and functions in a similar manner [2].

The philosophy of these groups is expressed in the Twelve Steps, a group of principles intended to be practised as a way of life. These 12 Steps include: admitting having a problem, searching for help, engaging in a thorough self-examination, making amends for harm done to others, and helping other drug addicts to recover. The central theme in these steps is a 'spiritual awakening'. Each member of the group is encouraged to cultivate an individual understanding (religious or non-religious) of this 'spiritual awakening'. The primary service provided is the group meetings. Members are encouraged to abstain completely from all drugs, and they share their successes and challenges in overcoming active addiction and living drug-free lives through applying the principles contained within the 12 Steps.

Historically, researchers and professionals have viewed practices involving the 12 Steps with a scepticism that some studies have supported. Kownacki et al. found in a meta-analysis of controlled studies that participants in AA meetings may sometimes do worse than non-participants [3]. However, the negative findings related to participants admitted into the groups by coercion.

Other studies have shown a clear connection between participation in a self-help group and a reduction in the use of drugs [4-6]. The positive effect of self-help groups is, amongst other things, attributed to a change of a social network [7]. Participants gain new abstinent friends and learn new coping strategies. Zemore et al. state that if you help others, you also help yourself. You increase involvement in your own recovery, achieve higher social status and build self-esteem [8].

In Scandinavian countries it is now becoming more common to find the 12-Step experiences integrated into standard addiction treatment. After the initial addiction treatment has been completed, patients are more often encouraged to participate in AA and NA groups to maintain their recovery. The only condition of participation is a desire to stop using drugs. There is, however, a lack of agreement about whether group participation has an independent effect, or whether the positive effect observed is due to selection biases [9].

The aim of our study was to monitor a group of patients who joined self-help groups after initial treatment, and to examine the correlation between participation, background variables and drug management. Our hypothesis was that participation in self-help groups increased the likelihood of continued abstinence.

The site of prior treatment was the Addiction Unit in Vest-Agder County, Norway. This unit accepts almost everyone who wants addiction treatment, without pre-selecting patients on the basis of socioeconomic or other criteria. The unit is a Hazelden-type treatment centre and the therapy given is grounded in the concept of alcohol and drug addiction as a spiritual and medical disease. The setting is in-patient treatment for a period of six weeks. In accordance with the 12-Step principles, the basic aim of the treatment is to achieve enduring abstinence. A major goal is also to foster the patient's commitment to participate in AA/NA, and patients are actively encouraged to attend meetings. The treatment is delivered by a multidisciplinary team including psychologists and psychiatrists, social workers, nurses, spiritual care professionals and substance abuse/addiction counsellors. The content of the intervention is consistent with the 12 Steps of AA/NA, with particular emphasis on the first five Steps. The centre uses a structured treatment scheme, which includes lectures about the mental structure of addiction/addictive way of thinking, and group therapy sessions. Family members are also invited into a psycho-educative family program over one week.

## Methods

The Addiction Unit at Sørlandet Hospital in Kristiansand is a public treatment institution that mainly recruits patients from Vest-Agder County (population 160,000). Everyone was encouraged to join an AA/NA group after treatment. One hundred and fourteen patients (79% of all patients admitted during the period 2001 to 2002) accepted this offer and began as group members. The remaining 21% of patients terminated their addiction treatments early for various reasons. All patients who completed the six week treatment course subsequently agreed to begin self-help group participation. All patients were more than 25 years old and had been diagnosed with an alcohol or drug dependency in accordance with ICD-10 [10]. The diagnosis was made by a psychiatrist, including clinical and psychiatric examination, and was supported by a SCID interview [11]. Fifty-nine patients, 18 women and 41 men, had the diagnosis F10.2 (alcohol dependency); 55 patients, 15 women and 40 men, had the diagnosis F19.2 (multiple drug dependency). The average age was 44 years for patients with alcohol dependency and 33 for patients with multiple drug dependency.

The department uses the *National Client Form for Addiction Treatment *on a regular basis [12]. This questionnaire contains 37 questions and was completed when the patients joined the self-help groups. It gathers information on socio-demographics, physical and psychological health and substance use.

The endpoints of the study were self-help group participation and abstinence. Freedom from drugs is defined in this study as total abstinence from all intoxicating drugs in line with the aim of the 12-step program.

The study was carried out two years after the patients started in the self-help groups, by which time six (5%) patients had died. Four of them had been diagnosed with alcohol dependency and two with multiple drug dependency. The causes of death are not known. These six patients were included in the group of non-respondents and also in the discontinuation analysis. The remaining 108 patients received a questionnaire with a selection of questions from the *National Client Form for Addiction Treatment *together with additional questions relating to their self-help group participation.

**Figure 1:**
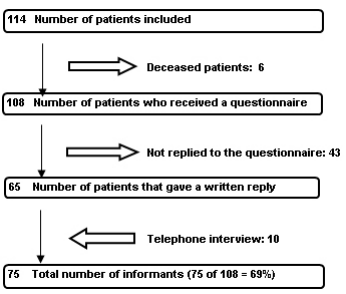
Flow chart for the follow-up study of self-help group participants.

The questionnaire was circulated between December 2003 and February 2004. Sixty-five replies were received after two reminders. During the final phase, a random selection of 20 of the non-respondents was called via telephone. Of these, 11 were successfully contacted and 10 were willing to participate. Each interview was conducted by the same person, and followed the structure set out in the questionnaire. Figure 1 shows the sequence of the study. The completed questionnaires were scanned with OCR equipment and analyzed using SPSS version 11.5.

The Regional Ethics Committee for Medical Research in Health Region South, Norway, waived the need for ethical approval for this routine follow-up questionnaire, as the questionnaire is a part of our standard procedure. All patients gave their informed consent when they were discharged from treatment and started in the self-help groups. The study has been reported to the Data Inspectorate of Norway, and was conducted in accordance with the Personal Data Regulations, 14 April, 2000.

### Statistical Methods

Cross tables on self-help group participation and abstinence were analyzed using Fisher's Exact Test for categorical variables and Student's t-test for continuous variables. A Logistic Regression Analysis (forward selection) was also performed. From bivariate analysis, variables with a P-value of less than 0.25 were included in the multivariate analysis. The significance level was set at p < 0.05.

## Results

Seventy-five patients (66% of the total population and 69% of those who had received a questionnaire) replied to the questionnaire. The response to the question on self-help groups was missing from one patient. Analysis of descriptive data from non-respondents showed no significant differences between respondents and non-respondents in respect of diagnosis, gender, age, accommodation status, cohabitation status or self-reported psychiatric conditions.

Two years after starting in the self-help groups, 43 patients (58%) still participated regularly (at least once a month). Using intention-to-treat-analysis, this is 38% of all 114 patients who initially enrolled in an AA/NA group, assuming that all non-respondents were non-participants or had died.

Table 1 compares the continuing group participants with non-participants. Both groups consisted of equal proportions of patients diagnosed with alcohol and multiple drug dependency. Single persons tended to participate more in self-help groups. A higher percentage of the non-participation group had received professional help for psychological problems, suffered from depression and/or attempted suicide. The only significant group difference was seen in the proportion of patients who had received medication for a psychological or emotional problem. Overall assessment indicates that the non-participating group suffered from greater psychological difficulties.

**Table 1:** Participation in self-help groups in the last six months (two years after starting).

	**Regular (n = 43)**	**Seldom or never (n = 31)**	**P-value**
Gender: % women	10 (23%)	11 (35%)	0.30
Age, years (SD)	38 (11)	37 (9)	0.67
Diagnosis:			
% F 19.2 (drug dependent)	20 (47%)	15 (48%)	1.00
% F 10.2 (alcohol dependent)	23 (53%)	16 (52%)	
Has injected drugs	17 (40%)	12 (39%)	1.00
Has previously been treated for drug misuse	32 (74%)	21 (68%)	0.60
Has suffered from serious depression (self reported)	24 (56%)	23 (74%)	0.15
Has suffered from serious anxiety (self reported)	20 (47%)	14 (45%)	1.00
Has attempted suicide	10 (23%)	13 (42%)	0.13
Has been given medication for a psychological/emotional problem	17 (40%)	24 (77%)	<0.01
Has received professional help for psychiatric problems	18 (42%)	20 (65%)	0.06
Social status:			
- No working income	26 (60%)	14 (45%)	0.25
- Cohabitant status single (n = 73)	35 (83%)	19 (61%)	0.06
- Homeless	8 (19%)	3 (10%)	0.34

A comparison of the background data between those who are regular participants (≥ once a month), and those who seldom or never participate (< once a month). The cross table analysis was conducted and the P-value was obtained with the Fishers Exact Test for categorical variables and the Student t-Test for continuous variables. The figures represent numbers and proportions (n = 74).

The results of a Logistic Regression Analysis suggested that cohabitation status 'single' and 'not prescribed psychiatric medicine' were the two strongest independent variables for continued group participation. The odds-ratio were 11.4 (95% CI; 2.4–55.0, p < 0.01) and 8.5 (95% CI; 2.1–32.8, p < 0.01) respectively.

Forty-four patients (59%) stated that they were abstinent two years after starting treatment (Table 2). One of these patients provided no information on her self-help group status. If we use intention-to-treat-analysis, the percentage of drug-free patients is 39%. The true percentage is probably between 39% and 59%.

**Table 2:** Drug use in the last six months (two years after starting) compared with background variables.

	**Not using any drug**	**Still using drugs**	**P-value**
	n = 44	n = 31	

Gender: % women	12 (27%)	10 (32%)	0.80
Age, years (SD)	39 (11)	37 (10)	0.43
Diagnosis:			
% F 19.2 (drug dependent)	17 (39%)	18 (58%)	0.11
% F 10.2 (alcohol dependent)	27 (61%)	13 (42%)	
Has injected drugs	17 (39%)	13 (42%)	0.63
Has previously been treated for drug misuse	31 (70%)	22 (71%)	1.00
Has suffered from serious depression (self reported)	27 (61%)	21 (68%)	0.63
Has suffered from serious anxiety (self reported)	20 (45%)	14 (45%)	1.00
Has attempted suicide	12 (27%)	12 (39%)	0.45
Has been given medication for a psychological/emotional problem	23 (52%)	19 (61%)	0.49
Has received professional help for psychiatric problems	25 (57%)	14 (45)%	0.48
Social status:			
- No working income	25 (57%)	16 (52%)	0.81
- Cohabitant status single (n = 73)	34 (77%)	21 (68%)	0.42
- Homeless	9 (21%)	2 (7%)	0.11
Self-help group participation (n = 74):			
- regularly	35 (81%)	8 (26%)	<0.001
- seldom/never	8 (19%)	23 (74%)	

The cross table analysis was conducted and the P-value was obtained with the Fishers Exact Test for categorical variables and the Student t-Test for continuous variables. The figures represent numbers and proportions (n = 75).

Table 2 shows a significant correlation between regular participation in a self-help group and a drug-free state. There was a tendency for the alcohol dependent and those who were previously homeless to manage better, but this was not significant. A Logistic Regression Analysis showed that regular participation in self-help groups was the only significant variable (OR 12.6; 95% CI; 4.1 – 38.3, p < 0.001). Consequently, the odds for a drug-free state were 12.6 times higher for those who participated regularly in the self-help groups than for those who did not.

## Discussion

The main finding in this study is that the number of patients remaining abstinent after two years is high. A self-reported drug-free state of 39–59% over the past six months, two years after start-up, is a good result. For comparison, Project Match had 36% during the past three months after three years [13]. At the same time, the proportion of abstinent patients is obviously greater among regular self-help group participants than among non-participants (81% versus 26%), and participation in a self-help group was the only predictive factor for staying abstinent. These findings are also supported by other studies [6,7], but are surprisingly marked in this study.

There is a tendency for those with alcohol dependency to have a better prognosis than those with multiple drug dependency. This has also been shown previously [14].

If we use intention-to-treat-analysis, 38% still participate in the groups. However, the 'true' proportion is probably somewhere between 38% and 58%. Of those who were interviewed over the telephone (after initially not having replied), three out of ten (30%) had participated during the past six months. Therefore, it is unlikely that all non-respondents had completely stopped participating.

In the Project Match study [13], subjects were randomized to Cognitive-Behavioural Treatment (CBT), Motivational Enhancement Therapy (MET) or Twelve Step Facilitation (TSF). One year after treatment, overall retention in self-help programs for the three interventions combined was 30 %. However, of the three types of intervention, it was mainly the 12-Step modalities that gave a recommendation to participate in a self-help program as a part of long-term rehabilitation strategy. Clear professional recommendations to facilitate self-help group involvement were rarer in the other types of interventions. Other studies have shown that the proportion of participants is higher in groups of patients who have exclusively completed the 12-Step treatment. Hoffmann, for example, found that 55% participated after one year [6]. He did not use intention-to-treat-analysis, and therefore his result should be compared with our 58% after two years.

It is possible that the self-help groups selectively retain individuals with particularly favourable prognoses. In assessing this possibility, it is important to consider the drop-out from the groups and whether the non-respondents conceal a negative selection. There is little in the background data to indicate that the patients who attended the groups had fewer problems with regard to severity of drug addiction, social status or accommodation situation. There is a non-significant tendency for those with high participation to have no income, and single status is a significant predictor. This tendency is supported by several studies indicating that those who are socially strained or are heavy drug addicts feel comfortable in the self-help groups [15,16].

Patients who stopped participating in the self-help groups had somewhat more severe psychiatric conditions. A significantly higher number of these patients had received medication for psychological/emotional problems and had previously received more professional help for such problems. A study from Iceland showed that a diagnosis of schizophrenia, but no other psychiatric co-morbidity, was negatively associated with participation in self-help groups [17]. If psychological difficulties result in drop-outs, this probably indicates limitations in the initiative of the target group, and implies that those with psychological difficulties need different kinds of help and should receive more social training and be eased into the group. Mueser et al. have suggested how this can be achieved [18].

### Limitations

The study has several limitations and methodical difficulties. It is a naturalistic study without a control group, and outcome data for such studies are often difficult to interpret. A positive correlation between AA/NA participation and a drug-free state need not necessarily indicate that AA/NA "works". Tournier has pointed out that the correlation only indicates "that alcoholics already committed to maintaining sobriety may gravitate toward AA to sustain their recovery" [19]. This raises the question of self-selection and suggests the alternative hypothesis that reduced drug abuse causes AA/NA affiliation. In other words, drug users who relapse tend to drop out of AA/NA, whereas those who are abstinent are more comfortable in continuing to attend meetings. There is also the question of whether anything in the group configuration predicts retention or relapse, for example whether the groups throw people out when they relapse. As far as we know, there are no formal obstacles to rejoining the groups after a relapse, and attendants are welcomed back at any stage. But we have no evidence to rule out the possibility that there are emotional and psychological obstacles to rejoining the groups. The problem of self-selection has been thoroughly discussed by other authors, and different statistical techniques have been used to address it [9,20]. In spite of these efforts, the positive correlation they observed could not be explained by the self-selection hypothesis.

We therefore find it difficult to ignore the clear difference in our study between those who participated in the groups and those who did not. It seems that no other background variables are able to explain the observed differences in abstinence. However, we acknowledge that hidden factors could have had an effect. We have not looked at possible major life events (establishing of family, negative health consequences etc.), which Gjeruldsen et al. found were important factors in stopping drug abuse [21], and we have not seen to what extent the respondents received professional help [17].

Another question is whether the selection from the Addiction Unit is representative of the entire population of drug addicts in the catchment area. The question is whether patients who are resistant to being treated in a 12-Step facility went somewhere else for treatment, or dropped out early. However, the institution receives almost all applications for treatment in the county, and we have no reason to believe that there is a significant selection of this type.

Other possible limitations to the study are implicit in the use of a questionnaire that was not validated. However, the form used has been incorporated nationally into the drug sector for the registration of background data of patients for several years, and has been regularly adjusted to optimize registration.

## Conclusion

This study shows a positive relationship between participation in 12-Step-based self-help groups and desired treatment results. A regression analysis of the background variables is unable to explain the differences found. This corroborates previous research in which self-help group participation and abstinence were found to be positively correlated. Sexton [22] also found a clear difference in the use of self-help groups between patients from institutions that explicitly advised participation and patients from institutions without this awareness. Health workers therefore ought to recommend their patients to participate in self-help groups as a part of their rehabilitation. However, the finding that psychological difficulties are associated with drop-outs suggests that self-help groups managed in a similar manner to Alcoholics Anonymous and Narcotics Anonymous may not be appropriate for everybody.

## Competing interests

The author(s) declare that they have no competing interests.

## Authors' contributions

The authors contributed equally to this work.

## Pre-publication history

The pre-publication history for this paper can be accessed here:



## References

[B1] Anonymous A (2006). Alcoholics Anonymous.

[B2] Anonymous N (2006). Narcotics Anonymous.

[B3] Kownacki RJ, Shadish WR (1999). Does Alcoholics Anonymous work? The results from a meta-analysis of controlled experiments. Subst Use Misuse.

[B4] Connors GJ, Tonigan JS, Miller WR (2001). A longitudinal model of intake symptomatology, AA participation and outcome: retrospective study of the project MATCH outpatient and aftercare samples. J Stud Alcohol.

[B5] Moos RH, Moos BS (2004). Long-term influence of duration and frequency of participation in Alcoholics Anonymous on individuals with alcohol use disorders. J Consult Clin Psychol.

[B6] Hoffmann NG, Harrison PA, Belille CA (1983). Alcoholics Anonymous after treatment: attendance and abstinence. Int J Addict.

[B7] Kaskutas LA, Bond J, Humphreys K (2002). Social networks as mediators of the effect of Alcoholics Anonymous. Addiction.

[B8] Zemore SE, Kaskutas LA, Ammon LN (2004). In 12-step groups, helping helps the helper. Addiction.

[B9] McKellar J, Stewart E, Humphreys K (2003). Alcoholics Anonymous involvement and positive alcohol-related outcomes: cause, consequence, or just a correlate? A prospective 2-year study of 2,319 alcohol-dependent men. J Consult Clin Psychol.

[B10] Organization WH (1993). The ICD-10 classification of mental and behavioural disorders diagnostic criteria for research.

[B11] MB. F (1997). Structured clinical interview for DSM-IV Axis I disorders (SCID-I) clinician version.

[B12] Lauritzen H, Astrid S (2004). Det nasjonale dokumentasjonssystemet innen tiltaksapparatet for rusmiddelmisbrukere.

[B13] group PM (1998). Matching alcoholism treatments to client heterogeneity: Project Match three-year drinking outcomes.. Alcohol Clin Exp Res.

[B14] Hoffmann NG, Miller NS (1993). Perspectives of effective treatment for alcohol and drug disorders. Psychiatr Clin North Am.

[B15] Brown BS, O'Grady KE, Farrell EV, Flechner IS, Nurco DN (2001). Factors associated with frequency of 12-Step attendance by drug abuse clients. Am J Drug Alcohol Abuse.

[B16] Humphreys K, Mavis B, Stofflemayr B (1991). Factors predicting attendance at self-help groups after substance abuse treatment: preliminary findings. J Consult Clin Psychol.

[B17] Tomasson K, Vaglum P (1998). Psychiatric co-morbidity and aftercare among alcoholics: a prospective study of a nationwide representative sample. Addiction.

[B18] KT M, DL N, RE D, L F (2003). Integrated treatment for dual disorders: a guide to effective practice.

[B19] Tournier RE (1979). Alcoholics Anonymous as treatment and as ideology. J Stud Alcohol.

[B20] Humphreys K (1996). Addressing self-selection effects in evaluations of mutual help groups and professional mental health services: an introduction to two-stage sample selection models. Evaluation and Program Planning.

[B21] Gjeruldsen S, Myrvang B, Opjordsmoen S (2003). Risk factors for drug addiction and its outcome. A follow-up study over 25 years. Nord J Psychiatry.

[B22] Sexton H (1995). Alkoholmisbrukere etter klinikkbehandling - en oppfølgingsundersøkelse.. Tidsskr Nor Lægeforen.

